# Novel, thalidomide-like, non-cereblon binding drug tetrafluorobornylphthalimide mitigates inflammation and brain injury

**DOI:** 10.1186/s12929-023-00907-5

**Published:** 2023-03-06

**Authors:** Daniela Lecca, Shih-Chang Hsueh, Weiming Luo, David Tweedie, Dong Seok Kim, Abdul Mannan Baig, Neil Vargesson, Yu Kyung Kim, Inho Hwang, Sun Kim, Barry J. Hoffer, Yung-Hsiao Chiang, Nigel H. Greig

**Affiliations:** 1 Drug Design and Development Section, Translational Gerontology Branch, Intramural Research Program National Institute On Aging, NIH, Baltimore, MD 21224 USA; 2 Aevisbio Inc., Gaithersburg, MD 20878 USA; 3 Aevis Bio Inc., Daejeon, 34141 Republic of Korea; 4 Department of Biological and Biomedical Sciences, Aga Khan University, Karachi, 74800 Pakistan; 5 School of Medicine, Medical Sciences and Nutrition, Institute of Medical Sciences, University of Aberdeen, Aberdeen, AB25 2ZD Scotland, UK; 6 Department of Neurological Surgery, Case Western Reserve University School of Medicine, Cleveland, OH 44106 USA; 7 Neuroscience Research Center, Taipei Medical University, Taipei, 110 Taiwan; 8 Department of Neurosurgery, Taipei Medical University Hospital, Department of Surgery, School of Medicine, College of Medicine, Taipei Medical University, Taipei, 110 Taiwan

**Keywords:** Neuroinflammation, Thalidomide, Cereblon, Immunomodulatory imide drugs (IMiDs), Neurodegeneration, Microglia, Teratogenicity, Spalt like transcription factor 4 (SALL4)

## Abstract

**Background:**

Quelling microglial-induced excessive neuroinflammation is a potential treatment strategy across neurological disorders, including traumatic brain injury (TBI), and can be achieved by thalidomide-like drugs albeit this approved drug class is compromised by potential teratogenicity. Tetrafluorobornylphthalimide (TFBP) and tetrafluoronorbornylphthalimide (TFNBP) were generated to retain the core phthalimide structure of thalidomide immunomodulatory imide drug (IMiD) class. However, the classical glutarimide ring was replaced by a bridged ring structure. TFBP/TFNBP were hence designed to retain beneficial anti-inflammatory properties of IMiDs but, importantly, hinder cereblon binding that underlies the adverse action of thalidomide-like drugs.

**Methods:**

TFBP/TFNBP were synthesized and evaluated for cereblon binding and anti-inflammatory actions in human and rodent cell cultures. Teratogenic potential was assessed in chicken embryos, and in vivo anti-inflammatory actions in rodents challenged with either lipopolysaccharide (LPS) or controlled cortical impact (CCI) moderate traumatic brain injury (TBI). Molecular modeling was performed to provide insight into drug/cereblon binding interactions.

**Results:**

TFBP/TFNBP reduced markers of inflammation in mouse macrophage-like RAW264.7 cell cultures and in rodents challenged with LPS, lowering proinflammatory cytokines. Binding studies demonstrated minimal interaction with cereblon, with no resulting degradation of teratogenicity-associated transcription factor SALL4 or of teratogenicity in chicken embryo assays. To evaluate the biological relevance of its anti-inflammatory actions, two doses of TFBP were administered to mice at 1 and 24 h post-injury following CCI TBI. Compared to vehicle treatment, TFBP reduced TBI lesion size together with TBI-induction of an activated microglial phenotype, as evaluated by immunohistochemistry 2-weeks post-injury. Behavioral evaluations at 1- and 2-weeks post-injury demonstrated TFBP provided more rapid recovery of TBI-induced motor coordination and balance impairments, versus vehicle treated mice.

**Conclusion:**

TFBP and TFNBP represent a new class of thalidomide-like IMiDs that lower proinflammatory cytokine generation but lack binding to cereblon, the main teratogenicity-associated mechanism. This aspect makes TFBP and TFNBP potentially safer than classic IMiDs for clinical use. TFBP provides a strategy to mitigate excessive neuroinflammation associated with moderate severity TBI to, thereby, improve behavioral outcome measures and warrants further investigation in neurological disorders involving a neuroinflammatory component.

**Graphical Abstract:**

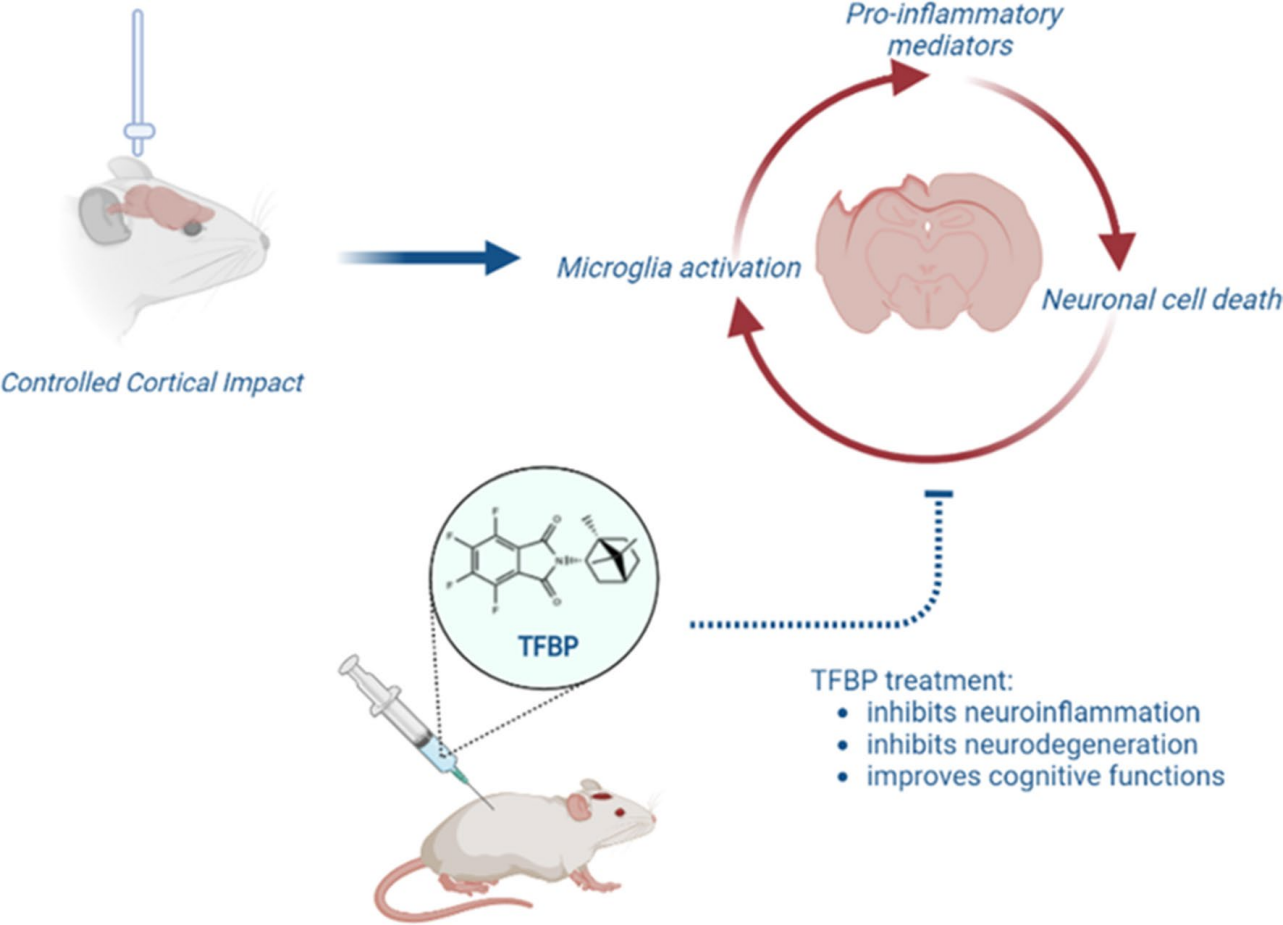

## Background

Traumatic brain injury (TBI), also known as the “silent epidemic”, is a common cause of disability and death worldwide, with a global annual incidence estimated between 64 and 74 million [1]. Although the majority of TBI cases involve mild concussions (approximately 80%), moderate and severe TBIs are characterized by a much higher mortality rate, particularly in older individuals [2]. Even in the case of TBI survival, recovery from injury is a long and challenging process. Indeed, moderate to severe TBI is commonly associated with the development of a number of symptoms that negatively impact the quality of life of the patient, and that involve physical, cognitive and behavioral sequela [3–5]. Additionally, TBI is increasingly recognized as a risk factor for the later development of neurodegenerative disorders such as Alzheimer’s disease (AD) and Parkinson’s disease (PD) [6–8], as well as neuropsychiatric conditions [9–12]. Currently, there is no pharmacological treatment approved for TBI, and it thus represents a disorder of significant new drug development need.

TBI neuropathology is typically described as the result of two major biochemical phases. The first is represented by the actual primary injury that results from an external mechanical force applied to the head, and is characterized by tissue damage, vascular injuries [including loss of the blood–brain barrier (BBB)] and diffuse axonal injury. Depending on the nature of the trauma, skull fracture may be present. These mechanisms largely result in necrotic cell death, which can be more or less substantial depending on the severity of the injury [13–15].

A long-lasting secondary phase of TBI consists of a series of biochemical cascades triggered by the primary trauma, and includes neuroinflammation, ischemia, mitochondrial dysfunction, glutamate excitotoxicity and hypoxia. These mechanisms ultimately result in synaptic dysfunction and apoptotic neuronal loss [14, 16–18]. Whereas the primary phase, consequent to its immediate nature, is not easily treatable, the biochemical mechanisms involved in the secondary stage of TBI may represent potentially druggable targets [19].

Over-excessive and chronic neuroinflammation, in particular, has been demonstrated to play a major role in the pathophysiology of TBI. Whereas an acute neuroinflammatory response is beneficial in instigating a reparative process after an injury, its excess and/or chronicity promotes a toxic cellular microenvironment that often ultimately translates into neuronal dysfunction and death [20–24].

Simplistically and as recently reviewed by Monsour and colleagues [21], in brain after TBI, microglia foster a pro-inflammatory environment. Similar to macrophages, microglia derive from myeloid precursor cells, and can further differentiate from monocytes into a pro-inflammatory M1 microglia phenotype or an anti-inflammatory/restorative M2 microglia form. Following TBI, the M1-like phenotype predominates (albeit a range of forms co-exist between the M1 and M2 phenotypes [25]) leading to a cascade of pro-inflammatory mediators, such as chemokines and cytokines [26–28]. These recruit further M1 microglia, amplify inflammation and generate chronic neuroinflammation. The accompanying release of reactive nitrogen and oxygen species, together with lipid peroxidation, mitochondrial and neuronal dysfunction can result in a loss of BBB integrity, and both induce peripheral inflammation [29] as well as recruit peripheral immune cells to the site brain injury [30].

Markers of activated microglial cells have been shown to be elevated shortly after TBI in preclinical models [31–33] and human studies [34–36]. One of the critical pro-inflammatory mediators whose levels are initially increased by activated glial cells is tumor necrosis factor (TNF)-α, which is widely considered a master regulator of the innate immune response. Animal and clinical studies have demonstrated an elevated presence of this cytokine following TBI [37, 38]. Targeting TNF-α has been proven to be beneficial in several models of TBI, resulting in a mitigation of neuronal and synaptic loss as well as improvement of the cognitive and/or motor functions in the injured animals [32, 39].

**Fig. 1:**
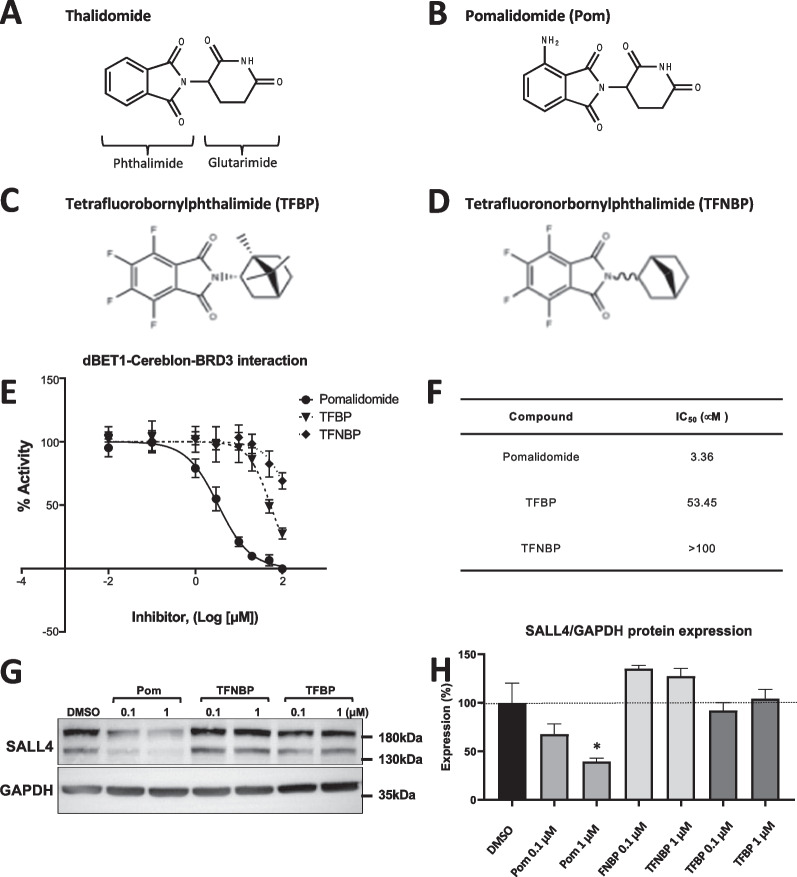
TFBP and TFNBP do not bind cereblon or lower levels of neo-substrate SALL4. Chemical structures of thalidomide (**A**) and pomalidomide (**B**). The binding of TFBP (**C**) and TFNBP (**D**) to cereblon was examined through use of a cereblon/BRD3 binding FRET assay (**E**). TFBP and TFNBP were not able to bind cereblon (IC_50_ 53.45 μM and > 100 μM, respectively), compared to Pom (IC_50_ 3.36 μM) (**F**). Concentration- dependent degradation of the downstream neo-substrates SALL4 was evaluated in human Tera-1 cells. TFBP and TFNBP at 0.1 μM and 1 μM did not lower expression levels of SALL4, in contrast to Pom at a 1 μM concentration (**G**, **H**). *p < 0.05 vs. control group. Values are presented as mean ± S.E.M., of n observations [N = 2 or 3 per group over 8 concentrations (**E**) to generate an IC_50_ value (**F**); N = 3 per group re: SALL4 expression (**H**)]

In this study, we evaluated the ability of two novel anti-inflammatory immunomodulatory imide drugs (IMiDs), tetrafluorobornylphthalimide (TFBP) and tetrafluoronorbornylphthalimide (TFNBP) (Fig. 1C, D), to mitigate pro-inflammatory signals in well-characterized cellular and animal inflammation models. Our prior studies with the clinically approved third generation IMiD, pomalidomide, demonstrated its ability to lower TNF-α generation and mitigate TBI-induced inflammation and behavioral impairments [32, 39], but its potential teratogenic actions remain a concern [40]. TFBP and TFNBP retain their phthalimide core structure of thalidomide-like drugs, with added fluorination to block potential phase 1 metabolic processes around this bicyclic ring. Importantly, the simple glutarimide ring of thalidomide and clinical analogues (Fig. 1A, B) is replaced by a more complex bridged ring, space occupying caged-like structure designed to sterically hinder potential binding with cereblon (Fig. 1C, D). Cereblon is a key target of thalidomide and analogues and forms a critical component of E3 ubiquitin ligase that tags the critical transcription factors Spalt Like Transcription Factor 4 (SALL4), Ikaros and Aiolos, for degradation [41]. The ubiquitination of these proteins, in large part, accounts for the antineoplastic (Ikaros and Aiolos) and teratogenicity (SALL4) of currently available IMiDs, but not necessarily their anti-inflammatory action. In this study, evaluation of TFBP demonstrated its lack of cereblon binding and downstream actions on SALL4. Evaluation in chick embryos demonstrated TFBP tolerability without obvious teratogenicity. Importantly, TFBP demonstrated ability to lower levels of lipopolysaccharide (LPS)-induced TNF-α and nitrite in RAW 264.7 cell cultures. The anti-inflammatory potential of this compound was then confirmed with in vivo studies, in a LPS model of both systemic and CNS inflammation as well as in a controlled cortical impact (CCI) model of moderate TBI.

## Methods

### Synthesis of the compounds

#### Synthesis of 4,5,6,7-tetrafluoro-2-((1R,2S,4R)-1,7,7-trimethylbicyclo [2.2.1]heptan-2-yl)isoindoline-1,3-dione (TFBP)

Mixtures of tetrafluorophthalic anhydride and (R)-(+)-bornylamine (equimolar) in acetic acid were stirred for 18 h under a nitrogen atmosphere at 120 °C (oil bath). Following removal of the solvent, the residues were first purified by silica gel chromatography (CH_2_Cl_2_), and then recrystallized twice with acetone to yield TFBP as white crystals. Chemical characterization was performed to confirm the structure.

#### Synthesis of 2-endo-(bicyclo[2.2.1]heptan-2-yl)-4,5,6,7-tetrafluoroisoindoline-1,3-dione (TFNBP)

Mixtures of tetrafluorophthalic anhydride, 2-aminonorbornane hydrochloride and triethylamine (equimolar) in acetic acid were stirred for 36.5 h under a nitrogen atmosphere at 100 °C (oil bath). After removing solvent, the residues were first purified by silica gel chromatography (CH_2_Cl_2_), and then recrystallized with acetone to yield TFNBP as white crystals. Chemical characterization was performed to confirm the structure.

### RAW 264.7 cell culture

RAW 264.7 mouse cells were purchased from ATCC (Manassas, VA, USA) and grown in DMEM media supplemented with 10% FCS, penicillin 100 U/ml and streptomycin 100 μg/ml (ThermoFisher Scientific, Asheville, NC, USA). Cells were maintained at 37 °C and 5% CO_2_, were propagated as described by ATCC guidelines, and cultured following the methods described in Tweedie et al., 2011 [42]. RAW 264.7 cells were treated with drug vehicle (DMSO, Sigma, St Louis, MO, USA) or the test compounds (TFBP or TFNBP, 100 nM to 1 µM), with 3–4 wells per treatment group (n = 3–4). One hour after the addition of the vehicle/test compounds the cells were challenged with LPS (30–60 ng/ml, *E. coli* O55:B5, Sigma, St Louis, MO, USA). Twenty-four hours later, the media was harvested and analyzed to determine cell viability and for the quantification of secreted TNF-α protein (ELISA MAX™ Deluxe Set Mouse TNF-α, BioLegend, CA, USA) and nitrite (Griess Reagent System, Promega, Madison, WI, USA). Cellular health was assessed by use of the CellTiter 96 AQueous One Solution Cell Proliferation Assay (Promega, Madison, WI, USA).

### Cereblon binding and neo-substrate degradation

A bead-based AlphaScreen technology was implemented for cereblon binding studies with nominal changes from the manufacturer’s protocol (BPS Bioscience catalog no. 79770). TFBP, TFNBP or pomalidomide were incubated with reaction mixtures that included cereblon/DNA damage-binding protein 1-Cullin 4a-ring-box protein 1 complex (CRBN/DDB1–CUL4A–Rbx1, 12.5 ng) and bromodomain-containing protein 3 (BRD3) (6.25 ng) in an Optiplate 384-well plate (PerkinElmer catalog no. 6007290). Following a half-hour incubation with shaking at room temperature, AlphaLISA anti-FLAG Acceptor and Alpha Glutathione Donor beads (PerkinElmer) were consecutively added and then incubated for a further hour at room temperature for each of the added chemicals. Alpha counts were subsequently read on a Synergy Neo2 (BioTek) for the analysis. The relative activity of the alpha signal was calculated after subtraction of the “blank value” from all readings and the value of vehicle group was then set as 100%.

The effect of TFBP activity on SALL4 was evaluated in Tera-1 cells. This cell line was obtained from the Korean Cell Line Bank (catalog no. 30105; Seoul, Korea) and grown in Dulbecco’s modified Eagle’s medium (DMEM) media, supplemented with 10% FBS, penicillin 100 U/mL and streptomycin 100 μg/mL, and maintained at 37 °C and 5% CO_2_. Tera-1 cells were treated with pomalidomide or TFBP or TFNBP (0.1 and 1 μM) for 4 h, and their cell lysates were prepared for the Western blot analysis, as described previously [43].

For Western blot analysis, total proteins were extracted using RIPA buffer (ThermoFisher Scientific, Waltham, MA) containing Halt Protease Inhibitor Cocktail (ThermoFisher Scientific). Next, proteins were separated by gel electrophoresis and then transferred to polyvinylidene difluoride (PVDF) membranes (ThermFisher Scientific), as described previously [43]. The following primary antibodies were used: (i) anti-SALL4 antibody (ab29112; 1:1000; Abcam, UK), and (ii) anti-GAPDH antibody (catalog no. ab8245; 1:5000; Abcam, UK). Subsequent to incubation at 4 °C overnight, the HRP-conjugated secondary goat anti-mouse IgG (ThermoFisher Scientific) was used to visualize SALL4 and GAPDH. GAPDH, a protein that is generally expressed across all eukaryotic cells, was used as an internal control to evaluate SALL4 protein expression levels. Antigen–antibody complexes were detected using enhanced chemiluminescence (ThermoFisher Scientific, iBright CL1500).

### Animal studies

All rodents were housed at 25 °C in a 12/12 h light/dark cycle with access to food and water ad libitum. The procedures used in this study were fully approved by the Institutional Animal Care and Use Committee of the Intramural Research Program, National Institute on Aging, NIH (detailed in approved animal protocols 331-TGB-2024; 488-TGB-2022). Studies were performed in accord of the ARRIVE guidelines and recommendations, and all efforts were undertaken to minimize any potential animal suffering and as well as the number of animals used. This was achieved by incorporating the outcome measures from our prior studies [44] and a power analysis [45]. In this first-in-animal evaluation of TFBP and TFNBP in a rodent model of LPS-induced systemic and neuroinflammation and of TFBP in CCI TBI, studies were conducted in male rodents alone to evaluate whether these novel agents demonstrate a signal of in vivo anti-inflammatory efficacy using the least number of animals possible. This decision was made in the knowledge that gender differences have been identified in the response of rodents to TBI, in addition to other brain insults [46–48]. In this regard, TBI incidence in young to middle-aged adults is lower in women than men, and derives primarily from different causes [46]. More confusing in human studies is the effect of gender on TBI outcome. Considerably fewer studies have focused on women challenged with TBI in relation to males, and outcome varies in relation to age, menopausal status as well as severity of TBI and chosen outcome measure. Whereas human studies often report worse outcomes in women than men, importantly animal studies largely describe the opposite [46]. In this regard, studies in ovariectomized rodents have demonstrated that estrogens provide significant neuroprotection to mitigate damage in female rodents, but not necessarily in the human species [46]. Hence, to avoid potential confounds associated with estrogen generation in young female rodents, or potentially ovariectomizing animals or aging them to a postmenopausal state, we performed our first-in-animal studies, reported herein, in young adult male rodents. Aware that there are undoubtedly gender differences in relation to the pharmacokinetics, pharmacodynamics and tolerability of TFBP and TFNBP, these could then be evaluated in future studies in the event that a promising signal of efficacy is demonstrated in the first-in-animal investigation described herein.

#### LPS model of inflammation in rats

Male Fischer 344 rats (Charles River Laboratories, Wilmington, MA, USA) (approx. 150 g weight) were randomly assigned across groups and, thereafter, administered either vehicle (Veh), or TFBP or TFNBP. Two doses of TFBPs were evaluated: (TFBP: 16.25 mg/kg or 32.5 mg/kg, and for TFNBP: 14.33 mg/kg or 28.66 mg/kg; i.e., equimolar doses), administered by the intraperitoneal (i.p.) route. These compounds were suspended in 1% carboxymethyl cellulose (CMC) in saline (0.9%), and administered 60 min prior to either administration of LPS (1 mg/kg, Sigma, St Louis, MO, *E.coli* O55:B5 in saline (0.9%), 0.1 ml/kg i.p.) or of Veh (CMC in saline (0.9%) 0.1 ml/kg, i.p.). The selected drug doses are, additionally, equimolar to that of pomalidomide (12.5 mg/kg and 25 mg/kg), which have been demonstrated to be well-tolerated in prior rodent studies, and are of translational relevance to humans [39]. The i.p. route of drug administration was selected for this first in animal study as it provides 100% bioavailability and, therefore, side steps any caveats associated with potential gastrointestinal bioavailability issues following oral administration.

At 4 h following LPS or Veh, animals were euthanized, and plasma and brain (cerebral cortex) tissue samples were collected and stored at − 80 °C. Brain samples were later sonicated in a Tris-based lysis buffer (Mesoscale Discovery, Gaithersburg, MD, USA) with protease/phosphatase inhibitors (Halt™ Protease and Phosphatase Inhibitor Cocktail, ThermoFisher Scientific, Asheville, NC, USA, diluted to 3X). Next, brain samples were centrifuged (10,000 g, 10 min, 4 °C), and protein concentrations were determined by Bicinchoninic acid assay (BCA, ThermoFisher Scientific, Asheville, NC, USA). An ELISA for TNF-α, IL-6, IL-1β, IL-10 and IL-13 was later performed following the manufacturer’s protocol (Mesoscale Discovery).

#### In vivo model of TBI

Mice were anesthetized with 2.5% tribromoethanol (Avertin: 250 mg/kg; Sigma, St. Louis, MO, USA) and placed in a stereotaxic frame (Kopf Instruments, Tujunga, CA, USA). A 4 mm craniotomy was performed using sterile procedures; the craniotomy point was selected midway between the lambda and bregma sutures and laterally midway between the central suture and the temporalis muscle. The skull fragment was then carefully removed without disruption of the underlying dura. Prior to injury induction, the tip of the impactor was angled and kept perpendicular to the exposed cortical surface. The mouse CCI instrument consisted of an electromagnetic impactor, Impact One (Leica Biosystems Inc., Buffalo Grove, IL, USA) that allows independent alteration of injury severity by controlling contact velocity and the level of cortical deformation. In these experiments, the contact velocity was set at 5.0 m/s, the dwell time was set at 0.2 s and the deformation depth was set at 2 mm to produce a moderate TBI. The injury site was allowed to dry prior to suturing the wound. During surgery and recovery, the body temperature of the animals was maintained at 36–37 °C by using a heating pad. Based on data generated from prior LPS experiments in rats where TFBP demonstrated greater efficacy compared to its close analogue TFNBP, TFBP was chosen for further investigations in the CCI model. Mice were randomized following CCI to treatment with either of two doses of TFBP (16.25 mg/kg and 32.5 mg/kg, i.p.) or saline, and were dosed at 1 and 24 h after the injury by the i.p. route.

#### Behavioral assessment for motor functions and coordination

Behavioral tests were performed 1 week and 2 weeks after injury, to assess changes in motor functions and coordination, compared to evaluations performed 1 week prior to CCI TBI. All tests were performed during the animals’ light phase; cages were transported to testing rooms at least 30 min prior to testing.

Beam Walking Test (BWT): A BWT was used to assess CCI-induced deficits in fine motor coordination. Mice have a preference for a darkened enclosed environment, as compared to an open illuminated one. Each animal was placed in darkened goal box for a 2 min habituation and then the trial began from the other (light) end of the beam. The beam was constructed with the following dimensions: 1.2 cm (width) × 91 cm (length). The time taken for each animal to traverse the beam to reach the dark goal box and the immobility time spent between the moment when they were initially placed on the beam and when they started walking were documented. Five trials were recorded for each animal before CCI and at 1 and 2 weeks after CCI. The mean times to traverse the beam and the immobility times were calculated, and a plot was generated to evaluate treatment effects; these times were then used for statistical analysis.

Gait analysis: For the gait analysis, mice were tested on a fixed-speed treadmill apparatus (DigiGait; Mouse Specifics). Mice were habituated to the apparatus for 1 min, and then given a 1-min run at 5 cm/s. Following a 1-min rest, the treadmill speed was increased to 15 cm/s. Video was collected at high speed from a ventrally placed camera, and 3–5 s of representative gait video was selected by an experienced but blinded user for automated analysis.

#### Tissue processing

Two weeks after injury, mice were deeply anesthetized with isoflurane and perfused transcardially with 30 ml phosphate buffered saline (PBS). After removal, the brain was post-fixed with 4% PFA overnight then transferred to a 30% sucrose solution in PBS. Coronal sections from the dorsal hippocampus and the posterior parietal cortex were cryosectioned at 25 μm thickness and stored in cryoprotectant solution for Giemsa staining and immunohistochemical analysis.

#### Quantification of brain lesion and lateral ventricle size in TBI animals

One set of brain section 2-weeks post-CCI were mounted on slides. The slices were then stained with 10% Giemsa KH_2_PO_4_ buffered solution (pH 4.5) for 30 min at 40 °C. After a brief rinse, slides were destained, differentiated, and dehydrated in absolute ethanol. Thereafter, the sections were cleared in xylene and then coverslipped. Brain image regions were quantified using ImageJ 1.52q software (National Institutes of Health, Bethesda, MD). The calculation formula for contusion volume size and lateral ventricle size were as follows: Σ (area of contralateral hemisphere − area of ipsilateral hemisphere)/Σ(area of contralateral hemisphere); Σ(area of ipsilateral lateral ventricle)/Σ(area of contralateral lateral ventricle). There were 9 brain sections from each mouse counted by blinded observers, with regions starting from bregma at 0.86 to − 1.46 mm.

#### Immunofluorescence analysis

For GFAP and Iba1 quantification, brain samples were incubated overnight with either one of the following primary antibodies: anti-GFAP 1:2500 dilution (chicken polyclonal, Abcam, USA, cat#ab4674) or anti-Iba1 1:200 dilution (guinea pig polyclonal anti-Iba1, Synaptic System, USA, cat#234004). After PBS washing, sections were incubated for 1 h at RT with a goat anti-chicken AlexaFluor^®^ 488-conjugated secondary antibody IgY (H + L) 1:500 dilution (ThermoFisher, USA, cat#A-21432) for detection of GFAP, and with a Goat anti-Guinea Pig IgG (H + L) Highly Cross-Adsorbed Secondary Antibody, Alexa Fluor™ 555, ThermoFisher, USA, cat#A-21435) for detection of Iba1. Sections were washed in PBS and mounted with VECTASHIELD^®^ Antifade Mounting Medium with DAPI (Vectorlabs, USA, cat#H-1200). Controls consisted of omission of the primary antibody.

Iba1 morphological analysis was performed on × 40 magnification images by using MotiQ, a plugin for Image J. MotiQ thresholder (v0.1.2) was used to create figures from immunofluorescences for the MotiQ analyser (v0.1.3). Multiple parameters were analyzed, including ramification index, spanned area, number of branches, junctions and endpoints.

#### Chick embryology and analysis

Chicken eggs were obtained from Henry Stewart & Co Ltd, Norfolk UK. All work with chicken embryos obeyed UK Home Office regulations and followed guidelines, standards and practices governed by the University of Aberdeen Ethics Committee (Scotland, UK). Each working solution of TFBP contained DMSO at 0.5% (3.5 μM TFBP), 1% (7.0 μM TFBP) and 2% (14.0 μM). Embryos were incubated at 37 °C for the required time period to reach E2.5 and E4 (early and mid-developmental stages, respectively). Eggs were then opened and the embryonic membranes protecting the embryos were removed with forceps. Chicken embryos typically lie on one side, so the left side is directly against the yolk and the right side can be observed. Drug (TFBP) or Control (DMSO alone) solutions were applied in 100 μL aliquots over the middle of the embryo on its right side. Embryos were left at room temperature for 20 min before being replaced in a 37 °C incubator. Due to the limited diffusion of drugs when applied to the right side of an embryo, the right side is considered the ‘treatment’ side and the left (facing internally towards the yolk sack) is considered normal after treatment and can, thereby, act as an internal control.

### Docking pockets and predictions of TFBP and thalidomide structural analogue interactions with cereblon

Assessments of prospective docking pockets and docking predictions for S enantiomeric forms of TFBP, TFNBP and thalidomide-like drugs on the structure of cereblon were investigated using automated software [49]. This was followed by a cavity-based blind drug docking prediction utility [50] to appraise the characteristics of the computed drug docking predictions of these IMiDs. The drug docking pockets and the binding differences between TFBP, TFNBP, thalidomide and pomalidomide in cereblon were determined for the best scoring attributes of these chemical agents. In short, the crystal structure of human cereblon in complex with DDB1 and lenalidomide (4TZ4: https://www.rcsb.org/structure/4TZ4) was downloaded in PDB format from the PDB database. The chain C (human cereblon) was separated from the remainder of the crystal structure complex and was utilized in docking predictions for the S enantiomeric forms of the study compounds. The S enantiomer was chosen as former x-ray crystallographic studies have reported that this enantiomer of thalidomide-like IMiDs better binds cereblon [51], notwithstanding that molecular modelling computational data does not necessarily simulate or fall in line with all experimental data from prior x-ray crystallographic studies [52]. Playmolecule, an automated server that employs a software DeepSite [48, 49] to establish the core binding sites, was used to simulate potential interactions between TFBP, TFNBP or thalidomide-like compounds with human cereblon. An automated docking software [53] was used to investigate potential similarities and differences in the pharmacophore pocket engaged by the test drugs. Briefly, two files were uploaded that included the C-chain (human cereblon) of the PDB ID 4TZ4 without lenalidomide and damaged DNA binding protein 1 (DDB1) and the drugs individually in their PDB formats to the Docking server [53]. For docking pocket predictions, the results appear as the number of preferential pockets determined with their relevant scores. Results of docking were collected with individual Vina scores, cavity sizes, docking centers, poses and sizes of predicted cavities for the drugs noted above. The resulting drug-cereblon complexes were visualized using the drug discovery studio visualizer software BIOVIA [49].

### Statistical analysis

Data were evaluated between groups with one-way analysis of variance (ANOVA) followed by Dunnett’s posthoc tests (GraphPad Prizm 7, San Diego, CA, USA) when appropriate for multiple comparisons. The behavioral data were evaluated between groups with two-way ANOVA followed by Dunnett’s posthoc test. Grubb’s test was used to identify and remove outliers. Bar graphs are presented as mean ± SEM values. A p value of < 0.05 was considered statistically significant, and levels of significance are provided in the legend for each individual Figure.

## Results

### TFBP and TFNBP did not bind cereblon or lower the expression of neo-substrate SALL4

Past studies have demonstrated that the primary teratogenic mechanism of thalidomide and analogues derive from their ability to bind cereblon and, thereby, elicit the ubiquitination and decreased expression of key downstream neo-substrates, particularly SALL4. SALL4 is recognized as the causal gene in the hereditary diseases Duane Radial Ray syndrome, Okihiro syndrome and Holt–Oram syndrome [54–56], which share several common characteristics with thalidomide embryopathy [57]. Considering the partial structural similarity of TFBP (1C) and TFNBP (1D) with thalidomide-analogue drugs (1A and 1B), we analyzed their interaction with cereblon through a cereblon/BRD3 binding FRET assay. TFBP and TFNBP were not able to bind cereblon (IC_50_ values of 53.45 µM and > 100 µM, respectively, that are in line with background binding values), compared to pomalidomide (IC_50_ 3.36 µM) (Fig. 1E, F). Whereas pomalidomide at 0.1 µM and 1 µM concentrations induced a significant decrease in expression levels of SALL4, treatment of human Tera-1 cells with TFBP and TFNBP did not affect this key neo-substrate (Fig. 1G, H).

### TFBP/ TFNBP and thalidomide structural analogue interactions with cereblon predict a lower binding affinity of TFBP/ TFNBP to the thalidomide binding pocket

**Fig. 2:**
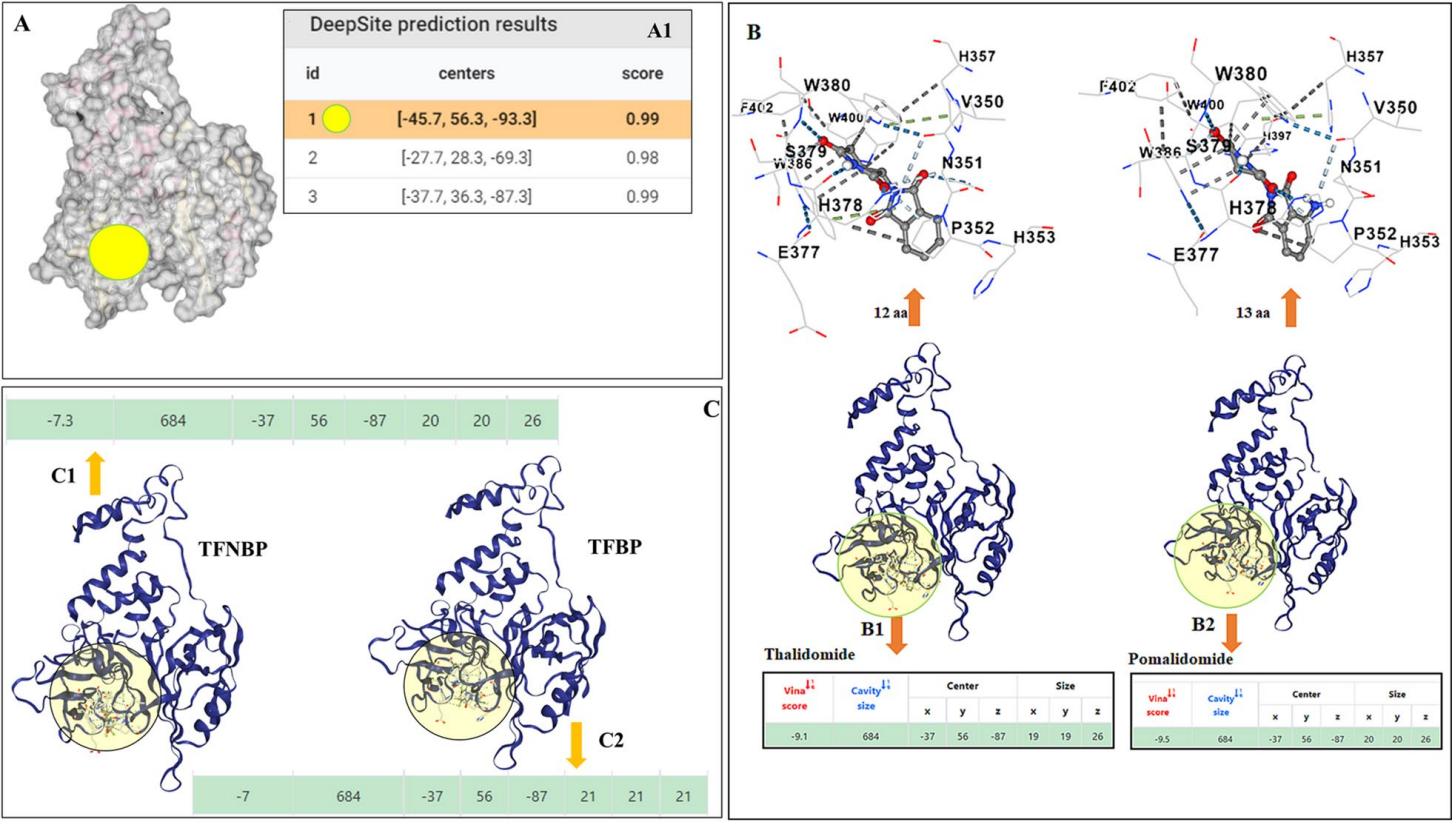
TFBP, TFNBP and IMiD drug docking pockets in chain C of human cereblon with the binding scores. (**A**) Determination of pockets for IMiD interactions within chain C of human cereblon showed the top 3 pharmacophores with their attributes (**A1**), with the pocket #1 (**A**, **B**, yellow circles) for binding the classic IMiDs thalidomide and pomalidomide (**B, B1, B2**). (**C**) The compounds TFNBP and TFBP docked with a lesser predicted preference at pocket #1 with scores (**C**-arrows and green strips) that demonstrated a substantially lower affinity than thalidomide and pomalidomide, which docked at pocket #1 with greatest preference and a higher binding affinity and associated Vina scores (**B**-green strips in insets). The binding pocket preferences and Vina scores of TFNBP and TFBP reflect a poor interaction probability of these compounds within the classic thalidomide binding pharmacophore (pocket #1)

To evaluate prospective interactions and the potential binding of TFBP, TFNBP and structural analogues of thalidomide with chain C of human cereblon and how this could contrast with the conventional IMiDs, molecular modeling studies were implemented utilizing the x-ray crystallographic structures of cereblon obtained from prior studies involving the IMiD lenalidomide (https://www.rcsb.org/structure/4TZ4). Pocket determination within cereblon forecast three top ranked pharmacophores (Fig. 2A–A1) with their attributes (Fig. 2A1). The docking prediction suggests that best thalidomide and pomalidomide binding/ interaction is for pocket number 1 (Fig. 2B, B1 and B2), which aligns with the pocket determined by x-ray crystallography studies [41, 51, 58, 59]. Vina scores were calculated as -9.1 and -9.5, respectively, for the S stereoisomer form of thalidomide and pomalidomide (Fig. 2, B1, B2 and insets), with the difference in scores arising from pomalidomide’s predicted interaction with more amino acids than thalidomide (Fig. 2B, B1, B2 upward directed arrows), allied to a better Vina score for pomalidomide. By contrast, TFNBP and TFBP were projected to occupy the same classic pharmacophore (pocket #1) but with Vina scores of − 7.3 and − 7.0, respectively (Fig. 2C, small green circle on the model). Notably, TFNBP and TFBP were predicted to also bind in proposed pockets #2 and #3 (Fig. 2A1).

These differences in docking pocket binding interactions of the evaluated IMiDs and, in particular, with the amino acids within the pharmacophore, not only determine the strength of binding interactions with cereblon but also the orientation of the IMID within the pocket and its potential binding to neo-substrates such as SALL4.

### TFBP does not induce overt teratogenicity-like changes when applied to chicken embryos

As an initial in vivo evaluation of teratogenicity, TFBP was applied to the right side of chicken embryos at an early (E2.5) and mid (E4) developmental stage, and embryos were examined at 24, 48 and 72 h, morphologically. As detailed in Table 1, three TFBP doses (3.5, 7.0 and 14.0 μM) were evaluated, together with DMSO alone. Whereas the lower TFBP dose proved to be well tolerated, the two higher doses resulted in an increasing death rate. No effects on embryo development were evident at the TFBP 3.5 and 7.0 μM doses or after DMSO alone; the deaths observed (TFBP 7.0 μM) appeared to be due to damage of the embryonic membranes that were removed before application of the drugs - as the embryos, themselves, appeared normal. A single embryo challenged with TFBP 14.0 μM had micro-opthalmia of the eye (Table 1). Notably, this was found in the eye facing away from the site of drug application (i.e., on the control side). No other developmental anomalies were evident, and the embryo was alive at the end of the experiment. In this light, the micro-opthalmia could potentially be a spontaneous malformation, as the three deaths seen at the 14.0 μM dose occurred quite late in development (around 48–72 h after drug application) and those embryos appeared developmentally and morphologically normal. All DMSO control embryos were normal.

**Table 1:** Dose and developmental stage-dependent actions of TFBP on chicken embryos

TFBP DOSE (μM)	AGE (days)							
	E2.5				E4			
	N	Normal	Abnormal	Dead	N	Normal	Abnormal	Dead
3.5	–	–	–	–	7	7	0	0
7.0	6	4	0	2	7	4	0	3
14.0	6	2	1*	3	–	–	–	–

TFBP topically was applied to early (E2.5) and mid (E4) developmental stage chicken embryos at several doses. Embryos were observed 24 h later and at every 24 h up to the end of the experiment at 72 h. Death rates increased as the dose of TFBP increased, likely due to drug-induced toxicity. At TFBP 3.5 and 7.0 μM doses, no effects on embryo development were seen; the deaths observed appeared to be due to damage of the embryonic membranes that were removed before application of the drugs, as the embryos themselves appeared normal. With the 14.0 μM dose, one embryo had micro-opthalmia of the eye (*), in the eye facing away from the site of drug application, but no other developmental anomalies (and was alive at the end of the experiment). The micro-opthalmia could thus potentially be a spontaneous malformation, as the 3 deaths seen at the 14 μM dose occurred quite late in development (around 48 and 72 h after drug application) and those embryos appeared developmentally and morphologically normal. All control embryos (DMSO; N = 2) were normal

### TFBP and TFNBP were well tolerated in cellular studies and mitigated elevated nitrite and TNF-α levels in RAW 264.7 mouse cells challenged with LPS

**Fig. 3:**
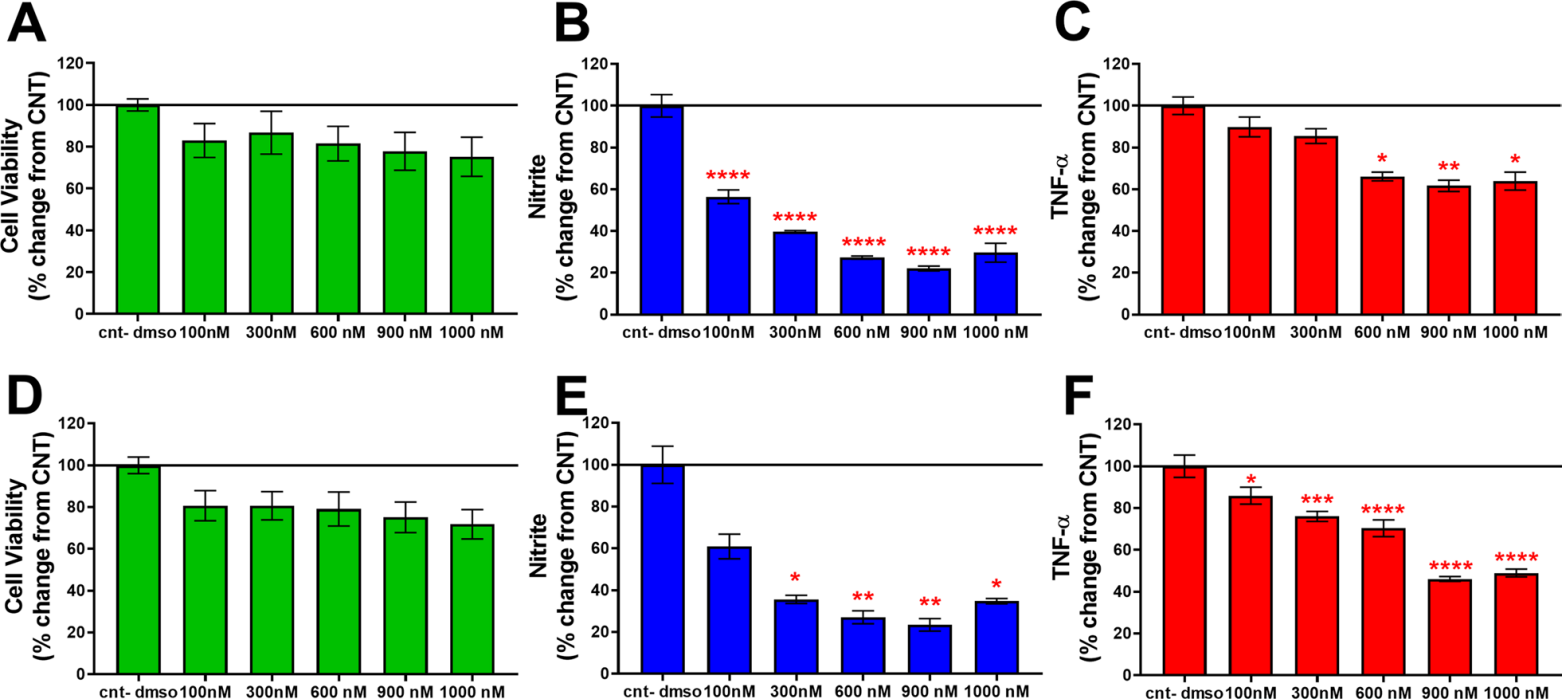
TFBP and TFNBP mitigate LPS-induced increases of nitrite and TNF-α in RAW 264.7 cell cultures. Challenge with LPS induced a spike in levels of nitrite and TNF-α, as compared to non-treated control cells (data not shown). Administration of TFBP reduced nitrite expression at the lowest evaluated 100 nM concentration (**B**), whereas elevated levels of TNF-α were mitigated starting at 600 nM (**C**). TFBP was well tolerated and was without impact on cell viability (**A**). Treatment with TFNBP was likewise effective in decreasing levels of nitrite and TNF-α, without affecting cell viability (Fig. 2D–F). *p < 0.05; **p < 0.01; ****p < 0.0001 vs. cnt-dmso group. N = 4 per group

Treatment of RAW 264.7 macrophage-like cell cultures with TFBP or TFNBP significantly decreased levels of LPS-induced nitrite and TNF-α in the cell culture media, without negatively impacting cell viability. Specifically, nitrite levels were significantly reduced starting at 100 nM concentration for TFBP and 300 nM for TFNBP, as compared to the LPS + vehicle group (cnt − dmso: Fig. 3B, E). TFBP’s action was less pronounced on TNF-α expression, but nevertheless induced significant TNF-α declines at concentrations equal to 600 nM and above (Fig. 3C). For TFNBP this was achieved at 300 nM and greater (Fig. 3F). Cell viability was not significantly affected by either agent across the concentrations evaluated (100–1000 nM), as compared to the LPS + vehicle group, (Fig. 3A, D); thereby indicating that noted reductions in nitrite and TNF-α were drug induced and not due to a loss of cell viability.

### TFBP and TFNBP significantly decreased levels of TNF-α, IFN-γ and IL-5 in plasma and cortex of LPS- challenged animals

**Fig. 4:**
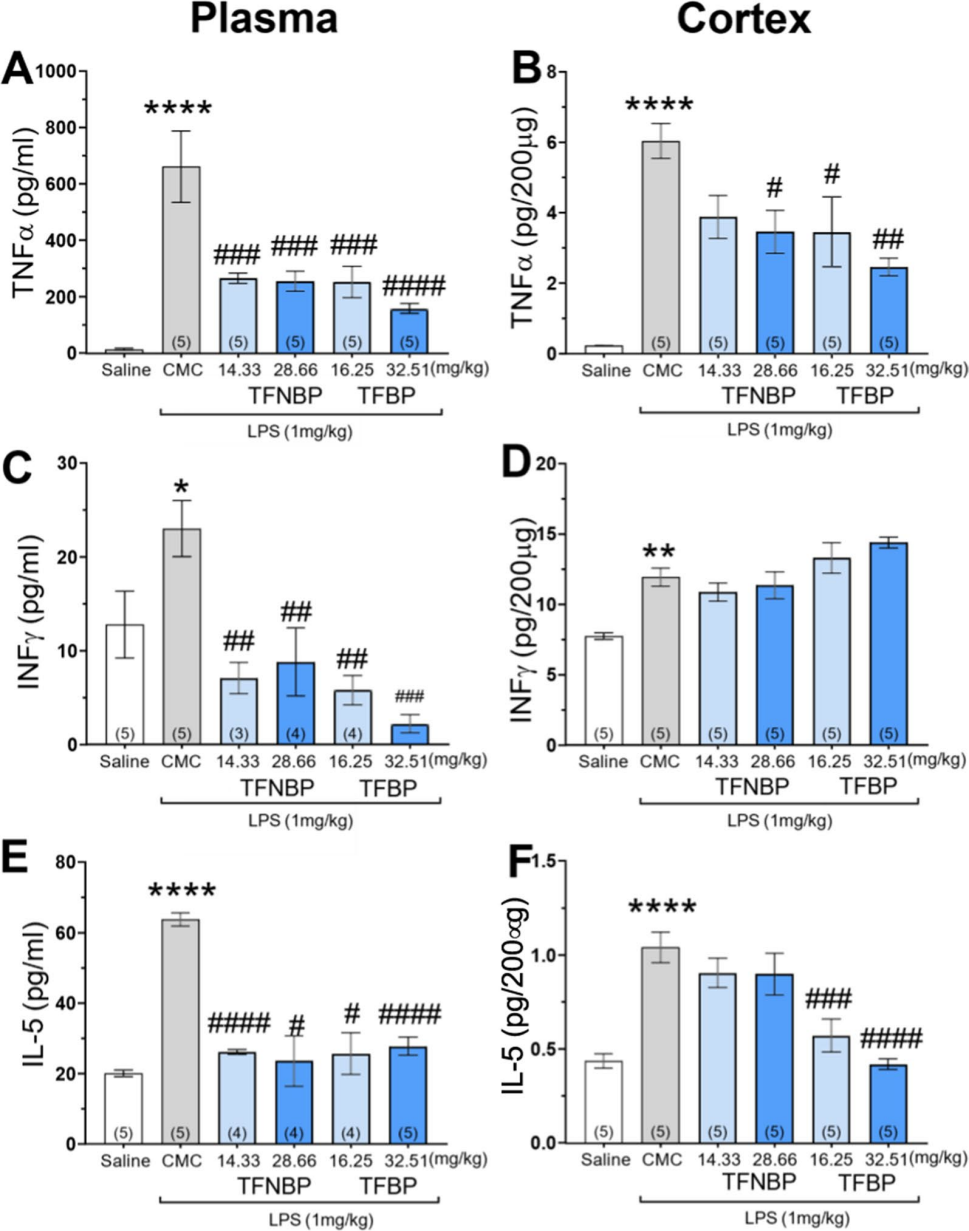
TFBP and TFNBP significantly decreased levels of TNF-α, IFN-γ and IL-5 in plasma and cortex of LPS- challenged animals. TFBP and its analogue TFNBP were evaluated in a LPS model of inflammation in rats (LPS 1 mg/ kg, i.p.). In this model, systemic administration of LPS induces elevations in pro-inflammatory proteins at 4 h in both plasma and brain (cerebral cortex). TFBP (formulated as a suspension in carboxymethyl cellulose (CMC) and administered i.p.) significantly decreased levels of pro-inflammatory cytokines (particularly TNF-α and IL-5) in both plasma and cortex, more significantly than TFNBP (**A**, **B**; **E**, **F**, respectively). A post-treatment reduction of IFN-γ was observed in plasma but not in cerebral cortex (**C**, **D**) On this basis, TFBP was selected for further in vivo investigation. *p < .05, **p < .01, ****p < .0001 vs saline control group. ^#^p < .05, ^##^p < .01, ^###^p < .001, ^####^p < .0001 vs LPS-treated group. ‘N value of animals’ shown at the base of each bar within brackets

As a first in vivo approach, we evaluated the ability of both TFBP and TFNBP to reduce LPS-mediated increases of proinflammatory cytokines in rats. Consistent with a previous study from our group [32, 43, 60], the systemic administration of LPS (1 mg/kg, i.p.) resulted in a substantial and statistically significant increment in TNF-α plasma levels, which were increased from 14.3 ± 6.4 to 661.2 ± 126.7 pg/ml, a 46-fold elevation at 4 h post LPS challenge (Fig. 4A), a time previously demonstrated to provide an approximate steady-state for TNF-α generation in response to LPS [42]. Both evaluated doses of TFBP and TFNBP reduced this increase (p < 0.001), with the higher dose of TFBP showing the greatest efficacy (decline: − 76% vs. LPS alone group, p < 0.0001). Cerebral cortex levels of TNF-α were, likewise, elevated in LPS-challenged animals, as compared to the control group without LPS (from 0.2 ± 0.02 to 6 ± 0.5 pg/200 μg, p < 0.0001, a 30-fold rise). Treatment with TFBP countered this increase more potently than TFNBP. Specifically, both low and high doses of TFBP were able to mitigate this rise (− 41.7% and − 58.3%, respectively), whereas TFNBP only effectively did so at the higher dose (− 41.7% vs. LPS group, Fig. 4B). Systemic LPS administration, additionally, elevated levels of IFN-γ in cerebral cortex, from 7.74 ± 0.25 pg/ml to 11.92 ± 0.63 pg/ml, as well as plasma (p = 0.053) (Fig. 4D and C, respectively); treatment with TFBNP and, more potently, with TFBP proved able to reduce this plasma rise [(Fig. 4C) p < 0.01 vs. control group for TFNBP low and high dose, as well as TFBP low dose; p < 0.001 for TFBP high dose vs. control]. Cerebral cortex IFN-γ levels were not impacted by either compound (Fig. 4D). Cortex and plasma levels of IL-5 were elevated following LPS challenge (p < 0.0001 vs control group) (Fig. 4E, F). Whereas TFBP reduced IL-5 expression in both tissues at both doses (p < 0.05 and p < 0.0001 vs. control group in plasma for low and high dose, respectively; p < 0.001 and p < 0.0001 vs. control group in cortex for low and high dose, respectively), TFNBP effectively did so in plasma, at both doses [(Fig. 4E) p < 0.0001 and p < 0.05 vs. control group for low and high dose, respectively], but not brain (Fig. 4F).

On the basis of the more effective mitigation of LPS-induced systemic and neuroinflammation provided by TFBP in comparison to equimolar TFNBP, the former was selected for evaluation of efficacy to counter a TBI challenge in rodents subjected to CCI. This TBI model has a well characterized neuroinflammatory component. Furthermore, it is associated with an early motor impairment that is evident at 1-week post-injury that gradually resolves over a subsequent week [44, 60].

### TFBP significantly mitigated TBI-induced motor function deficits in mice

**Fig. 5:**
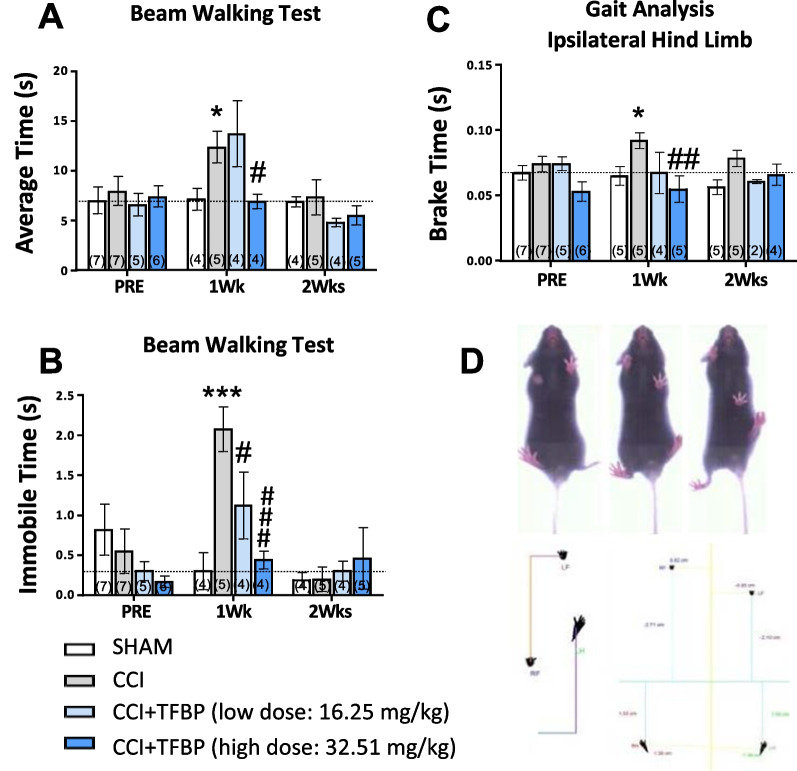
TFBP partially improved motor functions after TBI. In the beam walking test**,** CCI- challenged mice showed an increase in average time needed to traverse the beam (**A**), as well as in immobility time spent on the beam (**B**). TFBP (16.25 mg/kg and 32.5 mg/kg, i.p.), especially at the highest tested dose, mitigated this injury-induced increase, as seen in the behavioral assessment performed after 1 week. (**A**, **B**). Gait analysis was performed through DiGi Gait System (Mouse Specifics, Inc.). Treatment with TFBP (HD, high dose) countered the TBI-induced increase in brake time (time between initial paw contact to maximum paw contact), 1 week post injury (**C**). *p < 0.05, ***p < 0.001 vs control group; ^#^p < 0.05, ^##^p < 0.01, ^###^p < 0.001 vs CCI group). ‘N value of animals’ shown at the base of each bar within brackets

To evaluate the ability of TFBP to mitigate motor deficits induced by CCI injury, behavioral tests were performed at 1 week and 2 weeks after TBI, and were compared to similar ones performed 1 week prior to injury. The BWT was used as an assessment of motor coordination for TBI-challenged animals, by measuring (a) the average time that animals took to walk the platform, and (b) an immobility time that they spent at the platform starting point before beginning to walk. The CCI-alone (Veh) group exhibited an increase in both average transit time (p < 0.05 vs. sham group (without CCI)) and immobility time (p < 0.001 vs. sham group). TFBP at the higher tested dose prevented the CCI-induced behavioral impairment, as assessed by both measures (p < 0.05 for average transit time; p < 0.001 for immobility time). Treatment with the lower TFBP dose proved effective in mitigating the immobility measure (p < 0.05 vs. CCI-alone group), but not the average transit time deficit. As noted in a prior similar CCI study [60], deficits in BWT largely were resolved at 2-weeks. In this light, TFBP induced a more rapid recovery when evaluated a week after CCI, and no statistically significant differences were observed across groups at 2 weeks after injury (Fig. 5A, B).

The Gait analysis test was used to assess potential changes in spontaneous locomotion. Among the parameters analyzed by the DigiGait software, we observed that CCI-alone animals at 1 week showed an increased brake time, which represents the duration between the initial and the maximum paw contact, starting after the swing phase (p < 0.05 vs sham group). Treatment with the high dose of TFBP fully mitigated this rise (p < 0.001). No statistically significant differences were observed across groups at 2 weeks after CCI (Fig. 5C).

As TFBP demonstrated mitigation of motor deficits a week post CCI and functional deficits were not evident across groups at 2 weeks post injury, we interpret this as TFBP speeding spontaneous recovery of motor coordination following a TBI. To evaluate how this was achieved, immunohistochemical studies were subsequently undertaken to quantify the actions of TFBP vs. vehicle in relation to the TBI-induced lesion area and subsequent development of microglial mediated neuroinflammation.

### TFBP significantly decreased cortical lesion volume in CCI-challenged mice

**Fig. 6:**
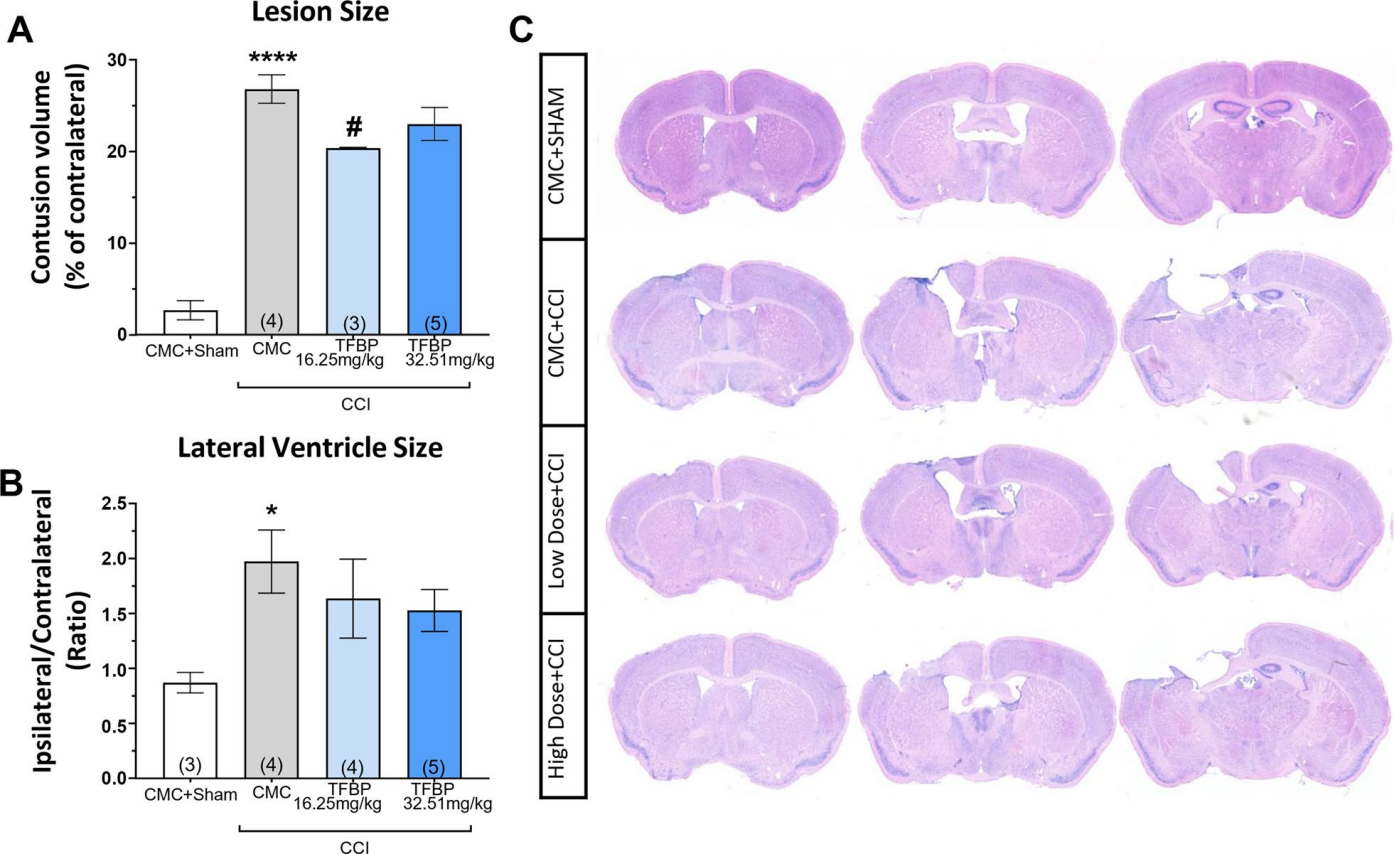
TFBP significantly decreased cortical lesion volume in CCI-challenged mice. CCI animals show loss of cortical tissue near the lesion site (**A**), as well as an enlargement of the lateral ventricle size. Treatment with TFBP (16.25 mg/kg and 32.5 mg/kg, i.p.) significantly reduced lesion volume induced by TBI; a similar trend is noticeable for the lateral ventricle size, although this does not reach statistical significance (**B**). Representative images of Giemsa-stained cortical sections (**C**). *p < 0.05, ****p < 0.0001 vs control; #p < 0.05 vs CCI). ‘N value of animals’ shown at the base of each bar within brackets

Giemsa histological staining was performed at 2 weeks after TBI to evaluate the contusion size and the lateral ventricle enlargement, which provides an indication of changes in intracranial cerebrospinal fluid (CSF). A direct result of the CCI procedure was a loss of cortical tissue around the TBI site, expressed as a percentage of the contralateral hemisphere (p < 0.0001 vs. sham). Treatment with the TFBP lower dose proved able to reduce loss of cortical tissue in CCI-challenged mice (p < 0.05 vs. CCI). A trend to decline was evident in the higher TFBP dose group that failed to reach statistical significance. An expansion in the size of the lateral ventricle was evident on the side ipsilateral to CCI injury in the CCI alone group [p < 0.05 vs. sham group (without CCI)]. A decreasing trend was noticeable in the TFBP treated groups in relation to this measure, but did not reach statistical significance (Fig. 6A, B).

### TFBP mitigates TBI-mediated expression of activated microglial cells

**Fig. 7:**
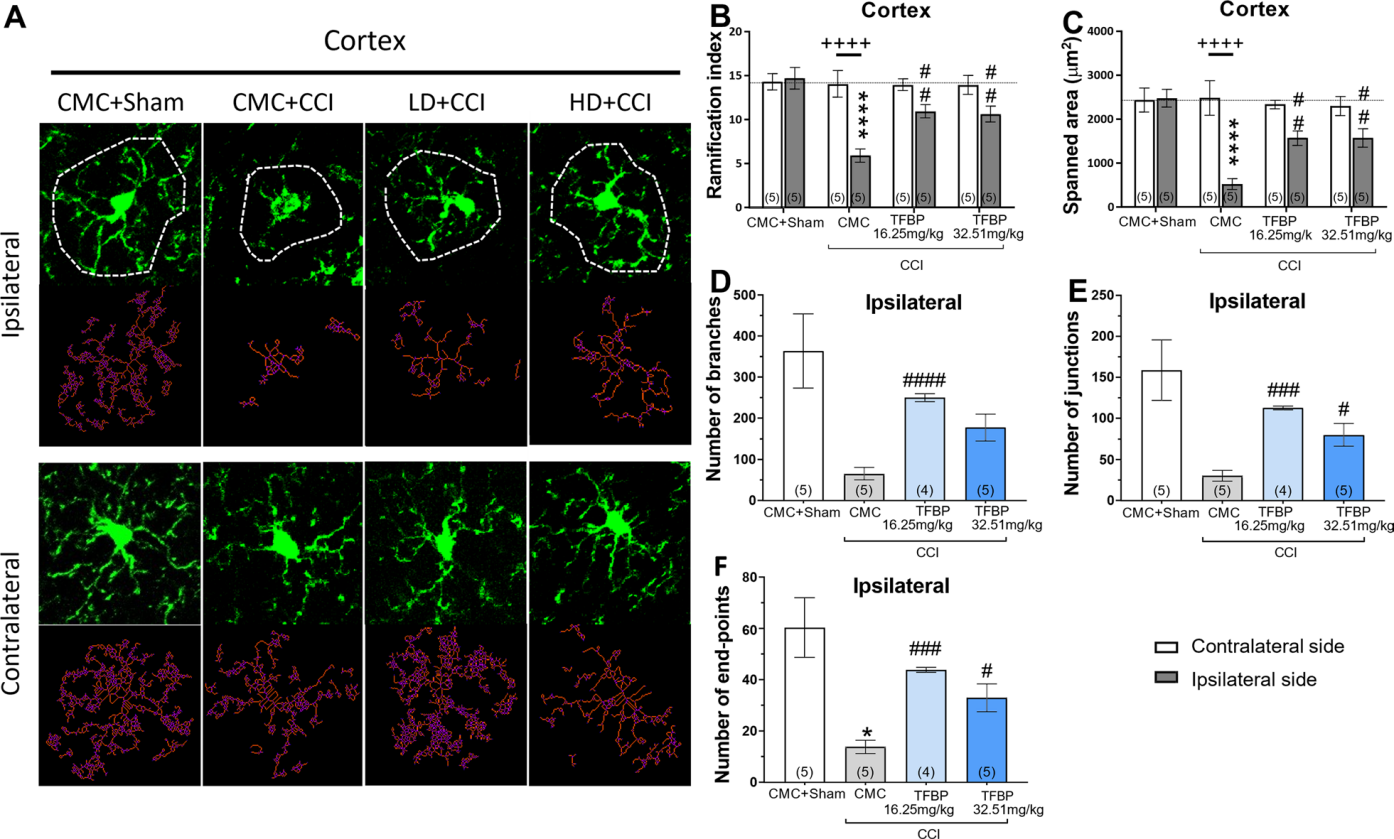
TFBP mitigates TBI-mediated expression of activation of microglial cells. CCI induces morphological changes in microglial cells (**A**), which are representative of an activated phenotype. Multiple parameters of Iba1 + cell morphology were analyzed, including ramification index (**B**), spanned area (**C**), number of branches (**D**), junctions (**E**) and endpoints (**F**). TFBP (16.25 mg/kg and 32.5 mg/kg, i.p.) mitigated the morphological changes induced by CCI in cerebral cortex, evaluated at 2 weeks post-injury (**B–F**). Representative images of Iba1 + cells at × 40 magnification and their skeleton reconstruction through MotiQ software (**A**). *p < 0.05, ****p < 0.0001 vs control group; ^#^p < 0.05, ^##^p < 0.01, ^###^p < 0.001, ^####^p < 0.0001 vs CCI group; + + + + p < 0.0001 vs Contralateral side. ‘N value of animals’ shown at the base of each bar within brackets

As neuroinflammation is a hallmark of CCI-induced TBI [31–33] and is associated with a change in microglial phenotype, MotiQ analysis with ImageJ software allowed us to evaluate multiple morphological parameters indicative of different microglial quiescent vs. activated phenotypes (Fig. 7A). Under physiological conditions, microglia adopt a ‘resting’ phenotype associated with the homeostatic physiological actions of microglia, characterized by a small soma and long and thin processes. In response to an insult, such as a CCI procedure, microglial cells begin to mediate an immune response by switching to an ‘activated’ proinflammatory state, morphologically characterized by an amoeboid shape, with an enlarged soma as well as thicker and shorter processes.

In comparison to sham control mice, microglia present in the cortical region ipsilateral to injury in the CCI-alone group expressed dramatic reductions in key morphological features characteristic of a resting (quiescent) phenotype, which included ramification index (Fig. 7B, p < 0.0001 vs. sham group), spanned area in µm^2^ (Fig. 7C, p < 0.0001 vs. sham group), number of branches (Fig. 7D), number of junctions (Fig. 7E), and number of end points (Fig. 7F, p < 0.05 vs. sham group). Notably, the contralateral side to the CCI lesion showed no statistically significant changes across groups in relation to these same morphological features (Fig. 7B, C; data not shown for the other parameters). Treatment with both the lower and higher dose of TFBP substantially counteracted these CCI-induced microglial phenotypic measures on the ipsilateral side, including ramification index and spanned area (p < 0.001 vs. sham group), as well as the number of end points (p < 0.001 and p < 0.05 for the low and high doses tested, respectively, vs. sham group) (Fig. 7D–F). A statistically significant reduction after treatment compared to the CCI group was also observed in relation to the number of branches (p < 0.0001 for TFBP low dose group, vs. CCI) and junctions (p < 0.001 for the low dose; p < 0.05 for the high dose, vs. CCI).

## Discussion

TBI is a leading cause of death and disability worldwide. Survivors of moderate to severe TBI often experience physical, behavioral and/or cognitive deficits that lead to serious consequences for them and their caregivers [5, 61], as there are no approved pharmacological treatments able to prevent development of long-term TBI symptoms. In this study, we evaluated novel IMiDs designed to lack cereblon binding. Finding that TFBP and TFNBP effectively mitigated key inflammation markers across in vitro and in vivo experimental models, including in brain, with TFBP demonstrating the greater anti-inflammatory action, we then evaluated TFBP in mice challenged with CCI-induced TBI—representative of moderate TBI in humans. Systemic TFBP administration reduced injury-associated neuronal cell death and decreased microglial activation; these neuroprotective/anti-inflammatory actions resulted in the more rapid mitigation of motor functional deficits induced by CCI injury. TBI impacts both genders across age [46, 47], making the potential use of classical IMiDs in women restrictive in the light of their known teratogenic actions, and impelled our evaluation of non-cereblon binding TFBP in a classical chicken embryo assay—in which a lack of teratogenic action was noted.

TBI neuropathology is commonly described as a 2-phase process. Little can be done to intervene pharmacologically during the first mechanical stage of the injury, but the secondary longer-lasting phase provides multiple potential therapeutic targets (neuroinflammation, excitotoxicity, mitochondrial dysfunction and axon degeneration [21, 26]). Neuroinflammation, when excessive and/or chronic, can play a major role in TBI development and progression [21–27]. Microglial activation occurs early post injury [31, 36]. CSF and brain pro-inflammatory cytokine levels, including TNF-α, IL-1β, IL-6, IFN-γ and IL-5, are reported elevated in TBI animal models and humans within hours of injury [62, 63]. Above all, TNF-α plays a key role in the ensuing TBI-mediated inflammation and is central in instigating initial glial cell activation [64–67]. This leads to amplification in the production and release of TNF-α as well as other inflammatory cytokines/mediators, including reactive oxygen species (ROS), nitric oxide, glutamate, and promotion of the complement cascade, which can worsen neuronal damage and induce cell death [63, 68, 69]. It is hence not surprising that many studies have focused on neuroinflammation as a potential therapeutic target in brain injury [21].

Anti-inflammatory strategies/targets vary widely, and although multiple anti-inflammatory drugs have demonstrated promise across TBI animal models [70, 71], fewer have reached human clinical trials where their results have been mixed [72]. Epidemiological studies of non-steroidal anti-inflammatory drugs (NSAIDs) suggest that such agents could be efficacious as a brain injury treatment approach [73–75]. However, NSAIDs have largely failed in randomized clinical trials [75]. Factors that may account for such failure are the multiple molecular pathways that regulate inflammatory status, and that their time-dependent profiles and outcomes may be different between preclinical animal models and humans. Initial drug design predicated on ‘target-based’ drug discovery can valuably identify and develop agents with ability to inhibit a pathway with high selectivity. However, when multiple parallel pathways exist in a pathological process, the selective action of the drug can potentially be by-passed. In contrast, our development of atypical IMiDs, such as TFBP, utilized a ‘phenotypic’ drug discovery approach that focuses on a biological action (e.g., mitigating inflammation) ‘agnostic’ to any target or mechanism to achieve this [76, 77]. Although both approaches (phenotypic- and target-based drug discovery) are valuable, the phenotypic approach generally provides more ‘first-in-class’ drugs, and is particularly advantageous when parallel mechanisms drive a pathological process, as very likely occurs in TBI and the induced inflammatory cascades. Different/parallel pathways may be more or less relevant in one individual as opposed to another, yet alone in one condition/ disorder vs. another, and thus one has more potential of efficacy using a drug with ability to hit several potentially useful targets, rather than a single target with huge selectivity.

The chemical structure of TFBP is characterized by the presence of a tetrafluorinated phthalimide group that provides these compounds the core element of IMiDs, such as thalidomide and analogues. One of the primary mechanisms through which this drug class exerts its anti-inflammatory effect is to lower TNF-α levels [78–80]. IMiDs interact with the 3’-untranslated region (UTR) of TNF-α mRNA, reducing the stability and half-life of TNF-α mRNA and, thereby, altering its transcriptional efficiency and ultimately lowering TNF-α protein levels [81, 82]. Thalidomide-analogues in TBI models provide an anti-inflammatory effect by mitigating glial activation and reducing the expression of not only TNF-α but also other key pro-inflammatory mediators [32, 39, 83]. In rodent CCI TBI, the thalidomide analogue 3,6’-dithiothalidomide (3,6’-DTT) inhibited microglial activation and downregulated TNF-α mRNA and protein levels at 8 h post injury [84]. More recently developed analogue 3,6′-dithiopomalidomide (3,6′-DP), as well pomalidomide, demonstrated comparable anti-inflammatory effects in a similar model of CCI. Both compounds reduced glial activation and expression of pro-inflammatory cytokines, including TNF-α, IL-1β and IL-6 [32, 39]. This was accompanied by a reduction of TBI-induced neuronal death, as evaluated by a decreased injury lesion volume, and an improvement of behavioral outcome scores [32, 39, 83].

Systemic and neuroinflammation are protective adaptive responses that can be activated by endogenous as well as exogenous stimuli, such as mis-folded proteins or LPS, respectively, and—when excessive or chronic—can lead to adverse health conditions, such as rheumatoid arthritis, atherosclerosis, metabolic disorders, and neurodegenerative disorders [85–87]. Two key inflammatory signaling cascades commonly triggered involve NF-κB and activator protein 1 (AP-1) pathways [88, 89], which can be stimulated by ligands to TNF-α receptors, IL-1 receptors as well as Toll-like receptors (TLRs). Activation of these pathways amplifies the expression of inflammatory cytokines as well as inducible nitric oxide synthase (iNOS), cyclooxygease-2 (COX-2) and other inflammatory factors. Additionally, AP-1 transcriptional activity provides regulatory input into cell survival, proliferation, and apoptosis [90, 91]. An increasing number of studies indicate that cereblon, a key drug target of IMiDs, has a regulatory role in inflammation via these two pathways and possibly others.

Cereblon is a substrate receptor of a E3 ubiquitin ligase, which comprises of damage-specific DNA-binding protein 1 (DDB1), cullin-4A/B, and the RING-box protein ROC1 [92–94]. This E3 ligase facilitates ubiquitination of endogenous substrates, exemplified by transcription regulator MEIS2, and when bound to thalidomide-like drugs modifies/hijacks cereblon’s substrate specificity to a different set of proteins that are then degraded (Ikaros and Aiolos: involved in IMiD efficacy in multiple myeloma, and by SALL4: involved in IMiD teratogenicity [41]). In parallel with such actions, cereblon can impact inflammatory signaling pathways through mechanisms both dependent and independent of its E3 ubiquitin ligase role. Regarding the former, Yang and colleagues [91] demonstrated that cereblon can ubiquitinate c-Jun to reduce its protein level and, thereby, attenuate the transcriptional activity of the AP-1 transcription factor complex, in which c-Jun represents an essential element and appears involved in brain injury [95]. In molecular studies involving human monocytic and murine macrophage cell lines challenged with LPS, with parallels to the cellular and in vivo LPS models evaluated in our study, Yang and colleagues established that cereblon lowered mRNA expression of pro-inflammatory cytokines downstream of the AP-1 signaling pathway and mitigated LPS-associated apoptosis [91]. In contrast, regarding E3 ubiquitin ligase independent action, Min and colleagues [96] reported that cereblon associates with the zinc finger domain of TRAF6, and decisively reduces ubiquitination of TRAF6 and TAB2; thereby lowering proinflammatory cytokine generation and NF-*κ*B-dependent gene expression. In line with this, cereblon overexpression leads to suppressed NF-*κ*B activation and lower pro-inflammatory levels in response to TLR4 stimulation with LPS, whereas cereblon knockdown results in a heightened pro-inflammatory response [96]. Classical thalidomide-like drugs can hence potentially induce anti-inflammatory actions mediated through cereblon E3 ubiquitin ligase-dependent and -independent mechanisms. Unlike thalidomide and pomalidomide, TFBP and TFNBP do not bind within the classical thalidomide binding domain (i.e., pocket #1) of human cereblon, as evaluated by cereblon/BRD3 binding FRET assay (Fig. 1E, F) and molecular modeling studies (Fig. 2C). Consequent to this, and unlike classical IMiDs, TFBP/TFNBP interactions with human cereblon do not support SALL4 degradation [evaluated by Western blot in human Tera-1 cells (Fig. 1G, H)], and thus anti-inflammatory action of these novel IMiDs is mediated via a cereblon independent mechanism in the absence of overt teratogenicity, as evaluated in TFBP challenged chicken embryo preliminary studies (Table 1) that previously have shown sensitivity to thalidomide [97]. In further support of this, key amino acid sequence differences exist between rodent and human cerebelon, with a particularly critical one occurring within the thalidomide binding domain (pocket #1). Whereas mouse cereblon is 95% homologous to the human form and can bind to thalidomide, degradation of SALL4 and related neo-substrates does not occur in the rodent and accounts for the lack of teratogenicity/antitumor action of classical IMiDs in rodents vs. their activity in humans (and chicken embryos), which can be conveyed to rodents by site-directed mutagenesis in the generation of cereblon-humanized mice [98, 99]. In contrast, thalidomide and conventional IMiDs induce anti-inflammatory actions in both wild-type and cereblon-humanized mice [98], likewise indicating presence of a cereblon-independent anti-inflammatory pathway.

In light of TFBP’s structural similarity to thalidomide-like IMiDs, we investigated its anti-inflammatory activity. As initial screening, we evaluated TFBP and its close analogue TFNBP in RAW 264.7 mouse cell cultures challenged with LPS. Both agents proved well tolerated and induced a dose-dependent decline in nitrite and TNF-α levels, two classical markers of inflammation whose expressions were markedly elevated by LPS. Anti-inflammatory activities of both TFBP and TFNBP were confirmed in an in vivo model of LPS inflammation, mitigating elevations in TNF-α and IL-5 in plasma and cerebral cortex, and IFN-γ in plasma. Their activity within the brain is in line with their calculated high CNS MPO (multiparameter optimization) scores of 3.4 and 4.3, for TFBP and TFNBP, respectively. Evaluating more potent TFBP in CCI TBI, quantification of neuroinflammation at 2 weeks post-injury demonstrated that TFBP post-TBI treatment mitigated microglial activation. Cell morphology evaluation showed that TFBP animals displayed a heightened expression of the microglial M2 phenotype, vs. the CCI-alone group, indicative of a mitigated inflammatory state. This TFBP anti-inflammatory effect was accompanied by a neuroprotective action, as observed as a reduction of injury lesion volume, and ultimately resulted in a more rapid recovery from motor and coordination impairments in CCI TBI-challenged mice.

A question arises as to why motoric behavioral changes found here largely resolve 2 weeks after CCI TBI, whereas the histological and biochemical changes in our study persist longer. There is much preclinical and clinical literature on compensatory central motor mechanisms after brain injury, involving both the ipsilateral hemisphere distal to the injury and the contralateral hemisphere. Although this literature is too extensive to be detailed here, the role of the contralateral hemisphere is most dramatically indicated by studies in the rodent split-brain preparation, where post injury motor compensation is eliminated after section of the corpus callosum [100]. Moreover, although the analogy is very clearly flawed and imprecise, 2 weeks in a mouse with an approximate 24-month lifespan could be considered roughly equivalent to approximately 70 weeks in a 70-year-old human. Interestingly in this regard, the clinical literature suggests there is significant resolution of some TBI sequelae at about 10–12 months after injury.

Another issue can involve the types of motor behavior analyzed here. Both ambulation on a narrow beam and walking on a treadmill involve balance as well as simple movement parameters. The clinical literature suggests that whereas simple walking may often recover within months of an injury, the recovery of balance, which requires both vestibular and limb proprioceptive function, is often delayed [101].

Summarizing, our in vitro and in vivo studies demonstrate a promising effect of TFBP in mitigating neuroinflammation and eliciting a protective action in a TBI model that aligns with moderate injury in humans. Notably, TFBP does not readily bind cereblon or trigger classical E3 ligase-mediated degradation of SALL4 that is considered central in the teratogenic adverse actions of classical thalidomide-like IMiDs [41, 102]. The present cellular, initial teratogenicity evaluation in chicken embryos, and first-in-rodent data demonstrate that TFBP is an interesting atypical novel IMiD that warrants further investigation in future male and female rodent studies as well as in further teratology investigations as a potential candidate drug for acute and chronic neurodegenerative conditions with an inflammatory component.

## Conclusions

The novel atypical IMiD TFBP, which lacks binding to cereblon, provides neuroprotective actions by reducing cortical neuronal loss and improving the behavioral outcome in a CCI mouse model of moderate TBI. Additionally, treatment with the compound mitigates injury-related changes in microglia morphology; the anti-inflammatory potential is also confirmed by its ability to reduce levels of pro-inflammatory cytokines in plasma and cerebral cortex in a classical LPS rat model of inflammation. As cereblon represents a target protein implicated in the teratogenicity of thalidomide-like drugs, TFBP warrants further preclinical evaluation as a candidate drug to potentially treat neurological and systemic disorders driven by an excessive inflammatory element.

## Institutional Review Board Statement

All rodent studies were carried out in accordance with the National Institutes of Health (DHEW publication 85–23, revised, 1996). Their use in research was approved by the Animal Care and Use Committee of the National Institute on Aging, NIH, Baltimore, MD, USA, Animal Care and Use Committee (approved protocol No. 331-TGB-2024 and488-TGB-2022). All work with chicken embryos obeyed UK Home Office regulations and followed guidelines, standards and practices governed by the University of Aberdeen Ethics Committee (Scotland, UK).

## Data Availability

Data is available from the corresponding authors on request.
